# Faster recovery and reduced paracetamol use – a meta-analysis of EPs 7630 in children with acute respiratory tract infections

**DOI:** 10.1186/s12887-019-1473-z

**Published:** 2019-04-23

**Authors:** Georg Seifert, Juliette Brandes-Schramm, Andrea Zimmermann, Walter Lehmacher, Wolfgang Kamin

**Affiliations:** 1 Charité - Universitätsmedizin Berlin, Clinic for Paediatrics, Augustenburger Platz 1, 13353 Berlin, Germany; 2 Clinical Research Department, Dr. Willmar Schwabe GmbH & Co. KG, Karlsruhe, Germany; 3 Emeritus -; University of Cologne, Institute of Medical Statistics, Informatics und Epidemiology, Cologne, Germany; 4 Clinic for Paediatrics, Evangelic Hospital Hamm, Hamm, Germany; 5 Faculty of Medicine, Pomeranian Medical University, Szczecin, Poland

**Keywords:** Pelargonium sidoides, Paracetamol, Upper respiratory tract infections, Children, Meta-analysis

## Abstract

**Objective:**

Fever is a very common adaptive immune response in acute respiratory tract disorders during infancy. Antipyretic / analgesic drugs such as paracetamol (acetaminophen) are widely used to improve the comfort of the child but may cause medically unneeded antipyresis and rare but potentially serious side effects. We assess whether treatment with *Pelargonium sidoides* extract EPs 7630 reduces the administration of paracetamol in children with acute tonsillopharyngitis (ATP) or acute bronchitis (AB).

**Design:**

Meta-analysis of randomised, placebo-controlled clinical trials.

**Methods:**

We searched clinical trial registries (ISRCTN, ClinicalTrials.gov) and medical literature (MEDLINE, EMBASE), for randomised, placebo-controlled trials investigating the administration of EPs 7630 to children with ATP or AB and reporting the co-administration of paracetamol. Based on the individual participant data of the eligible trials, study populations were characterized according to sex and age, and meta-analyses were performed for cumulative paracetamol use and ability to attend school at treatment end.

**Results:**

Six trials including a total of 523 children aged 6–10 years (EPs 7630: 265; placebo: 258) and suffering from non-β-hemolytic streptococcal ATP (3 trials) or from AB (3 trials) were identified and eligible. Children received EPs 7630 or placebo for 6 (ATP) or 7 days (AB). Compared to placebo, EPs 7630 reduced the cumulative dose of paracetamol in 5 out of the 6 trials, by an average of 244 mg (Hedges’ g; − 0.28; 95% confidence interval: [− 0.53; − 0.02]; *p* < 0.03). At treatment end, 30.2% (EPs 7630) and 74.4% (placebo) of the children were still unable to attend school (risk ratio: 0.43; 95% confidence interval: [0.29; 0.65]; *p* < 0.001).

**Conclusions:**

In children aged 6–10 years with AB or ATP, EPs 7630 alleviated the symptom burden and accelerated recovery. Although EPs 7630 has no known antipyretic effect, concomitant use of paracetamol was reduced.

## Background

Fever is among the most common symptoms in children consulting a paediatrician and has been estimated to account for about one third of all presenting conditions [1, 2]. Although the regulated increase in body temperature is metabolically costly to the organism, fever has been shaped and conserved over millions of years of natural selection because it confers a survival benefit in coping with inflammation and infection that outweighs its metabolic cost [3, 4]. From an immunological perspective, fever is an adaptive host response that has a direct antimicrobial effect and augments humoral and cellular defence mechanisms [4–7]: in vitro experiments show that fever plays a key role in reducing the virulence and increasing the clearance of microorganisms, as well as in stimulating the immune response by supporting the activation and proliferation of lymphocytes [4, 5], by enhancing the phagocytic potential of dendritic cells, and by augmenting INF-α production in response to viral infection [3, 5].

While there is no evidence that antipyresis reduces the duration of diseases [8], or that children with fever are at an increased risk of adverse outcomes [2], studies indicate overwhelmingly that the use of antipyretic drugs may prolong illness, may adversely affect clinical outcome during infection, and may even increase mortality in critically ill patients [4, 9–11]. Moreover, the administration of antipyretic drugs may mask important clinical signs of a patient’s condition [12].

In Western countries, the administration of over-the-counter (OTC) antipyretic drugs is very common, particularly in the paediatric setting [13]. Parents have been observed to administer antipyretics even in cases when there is only minimal or no fever [14], and as many as one half of parents were found to administer incorrect doses, with about 15% giving their children doses in the supra-therapeutic range [15]. A contributing factor to antipyretics overuse in children is the still widespread misconception of parents that fever is a disease rather than a symptom or sign of illness [1]. Fever is associated with discomfort and fatigue, and the administration of antipyretic drugs is mainly motivated by the desire to improve the overall comfort of the febrile child and the belief that feeling better is a direct result of the lowering of the fever [2, 5]. It is important to note, however, that the drugs most commonly used for antipyresis, such as paracetamol (acetaminophen), acetylsalicylic acid, and ibuprofen, not only have an antipyretic, but also an analgesic effect, and that their beneficial effect on the child’s well-being has been shown to be mainly attributable to their analgesic rather than to the antipyretic action [2, 4].

Acute respiratory tract infections (RTIs) are among the most common causes of fever in children [16, 17]. It is estimated that at least 90% of RTIs have a viral aetiology [18–20]. Acute tonsillopharyngitis (ATP) is a highly prevalent, seasonal infective disorder characterized by an inflammation of the pharynx and the palatine tonsils, which occurs in all age groups and accounts for about 5% of all visits in paediatric care [21]. Common symptoms of ATP include sore throat, dysphagia, red pharynx, enlarged tonsils covered with a yellow, blood-tinged exudate, fever with sudden onset, malaise, gastrointestinal complaints, halitosis, rhinorrhoea and cough [22]. More than 60% of all cases of ATP have a viral origin. Rhinovirus, coronavirus and adenovirus are the most common causes, but Epstein Barr virus, influenza and parainfluenza virus may also cause ATP [23]. Bacterial ATP is most commonly caused by group A streptococcus (GAS), whose prevalence in children of all ages with ATP has been estimated at 37%, with a lower prevalence in pre-school children [21]. Another very common RTI in children and adults is acute bronchitis (AB), a condition whose primary symptom is coughing, and which typically persists for about 3 weeks [24]. Since cough is also highly prevalent in other diseases such as allergic rhinitis, asthma, sinusitis, and the common cold, a differential diagnosis may be demanding. About 90% of all cases of AB are assumed to have a viral origin [25].

In an acute viral disease like ATP and AB, only minimal treatment emergent risk and side effects can be justified, notably in children. Unless there is evidence of bacterial infection (in which antibiotic treatment could be indicated), the focus of treatment is therefore on symptom control and accelerated recovery. In ATP, current disease management guidelines from Europe and North America agree that antibiotic treatment should only be administered in cases of confirmed bacterial infection, even in which some guidelines advocate the use of antibiotics only for patients who are at an increased risk of severe complications [26]. Supportive treatment, which is recommended in all other cases, is aimed at relieving the symptoms caused by ATP, and includes analgesia, hydration, and rest. In AB, the inappropriate use of antibiotics for infections of viral origin is equally discouraged, and instead the use of an antitussive medication (e. g., dextromethorphan, codeine, hydrocodone) is suggested [27]. Dextromethorphan in particular has, however, been shown to be inefficacious in children with AB [28], and treatment associated risks have prompted the American Academy of Paediatrics and the Food and Drug Administration (FDA) to recommend against the use of antitussive drugs in children under 6 years of age [24, 29]. For expectorants, no beneficial effect could be demonstrated in AB as well [30].

EPs 7630^1^ is an extract from the roots of *Pelargonium sidoides*. In acute respiratory infections such as ATP and AB, randomised, placebo-controlled trials have confirmed the symptom alleviating effect of EPs 7630 [31–35], while a comprehensive safety review based on more than 8000 patients exposed to EPs 7630 showed adverse event rates similar to those observed for placebo [36]. Pharmacological studies indicate that the clinical effects of EPs 7630 are closely related to its antiviral and antibacterial action. The extract supports the immune response of the organism by stimulating the release of tumour necrosis factor α, nitric oxides and interferon-β as well as by increasing the activity of natural killer cells [37–40]. Although EPs 7630 has no direct virucidal effect, antiviral activity is achieved through a reduction of the host cell infection attributable to an inhibition of virus replication, as shown for seasonal influenza A virus, respiratory syncytial virus, human coronavirus, parainfluenza virus, and coxsackie virus in vitro and in an animal model [41, 42]. Moreover, EPs 7630 was found to exert an antibacterial effect by inhibiting the adhesion of GAS to human epithelial cells [43–45].

Paracetamol is the most widely used medicinal product in children and has long been recognized as a safe and efficacious analgesic and antipyretic drug when used in the recommended dose range [46–49]. It may, however, cause inept antipyresis and potentially serious adverse effects [50–52]. In this paper we present the results of a meta-analysis of randomised, placebo-controlled trials performed to investigate whether treatment with EPs 7630 may reduce the co-administration of paracetamol in children suffering from ATP or AB.

## Methods

### Design, intervention, study eligibility criteria

A meta-analysis of clinical trials was performed to assess the effect of EPs 7630 on paracetamol administration in children suffering from ATP or AB.

EPs 7630 is an extract from the roots of *Pelargonium sidoides*, drug-extract ratio 1: 8–10, extraction solvent: ethanol 11% (*w*/w). The marketed medicinal product is available as a liquid solution, with a recommended daily dose of 3 × 10 drops for young children aged 1–5 years, 3 × 20 drops for children aged 6–12 years, and 3 × 30 drops for adolescents over 12 years and for adults, and as film-coated tablets containing 20 mg of extract, with a recommended daily dose of 3 × 1 tablet licensed for adolescents over 12 years and for adults. A syrup formulation for children aged 1–12 years is also available but was not used in any of the identified studies.

The analysis was performed using individual participant data from double-blind, randomised, placebo-controlled clinical trials with EPs 7630 which included children 6–10 years of age in one of the target indications. Eligible studies had to allow concomitant use of paracetamol and had to report the amount of the drug administered. No other restrictions applied.

Study selection and methods of the analysis were specified in advance and documented accordingly.

### Data sources and searches

Studies were identified from clinical trial registries (ISRCTN; ClinTrials.gov) and medical literature (MEDLINE, EMBASE), searched from the earliest record to the end of December 2016 and using the term ‘EPs 7630’ or ‘Umckaloabo’ in combination with ‘tonsillopharyngitis’, ‘sore throat’, ‘acute bronchitis’, or ‘cough’, as well as ‘placebo controlled’, for title, abstract, and keywords search. Identified studies were then examined for compliance with our remaining eligibility criteria.

### Outcomes

From the trials identified during the search, we extracted information regarding study methods and assessments. Data to be included into our analyses were patient age, sex, investigational treatment, paracetamol consumption, and inability to attend school due to ATP or AB.

### Data extraction, synthesis and analysis

All studies meeting the eligibility criteria were included into the analyses without further restrictions. Patient information was extracted from the raw data of the identified trials, which were made available by the study sponsor.

Sample characteristics were analysed using applicable descriptive summary measures. For cumulative paracetamol consumption (amount in mg) a meta-analysis was performed across all eligible trials by computing the difference between the mean values of the treatment groups and the associated 95% confidence intervals (CIs) in the original scale. Since the eligible trials used different prescriptions for paracetamol dosing (see details below), so that size effects could not be excluded, we used Hedges’ g as a standardised effect size measure for performing the meta-analysis [53]. Moreover, the percentage of children and adolescents who were unable to attend school at the end of the scheduled treatment period was entered into a meta-analysis based on risk ratios and their 95% CIs. Heterogeneity between the trials was assessed using the χ^2^-test for heterogeneity and the I^2^ statistic. Pooled meta-analysis estimates were obtained using random effects models throughout. Review Manager Version 5.2 software was used for all meta-analyses [54]. All specified *p*-values are two-sided. For meta-analyses, treatment differences were considered descriptively significant if the 95% CI of the point estimate did not include the value of 0 for differences or the value of 1 for risk ratios, corresponding to a descriptive p-value of *p* ≤ 0.05. All analyses were performed based on the full analysis set of study participants which was the primary data set for the analysis of efficacy in all original trials.

In patients terminating their study participation prematurely, cumulative paracetamol intake was calculated for the time period between the start of randomised treatment and early withdrawal. The analysis of the ability to attend school was based on the last available information.

## Results

### Search results, characteristics of included studies and participants

**Fig. 1:**
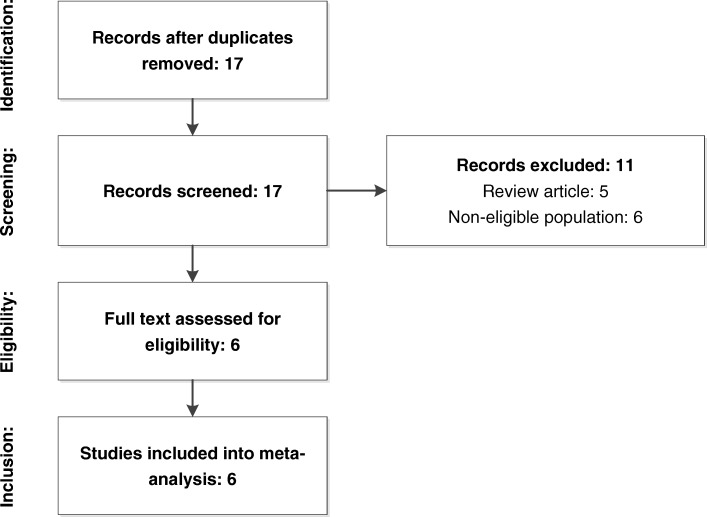
Search results and study selection

Our searches identified a total of 17 unique publications [31, 34, 55–69]. Five were review articles from which no additional original publications meeting our selection criteria could be inferred [31, 34, 61–63]. Five presented results from clinical trials with EPs 7630 in the indication of interest but did not include children 6–10 years of age [64–68]. One publication [69] reported on a pilot study with 28 participating children, but all participants were under 6 years of age, and the authors did not report on paracetamol consumption. The remaining 6 publications [55–60] met our eligibility criteria and were included into the meta-analysis (Fig. 1).

All eligible studies were double-blind, randomised, placebo-controlled trials published in peer-reviewed journals. Randomisation sequences were generated by independent statisticians using validated software. In all studies the investigational medicinal products were dispensed in sequentially numbered containers of identical appearance. Each participating subject was to receive the study medication corresponding to the lowest yet unassigned randomisation number available at the applicable centre. The studies were sponsored by Dr. Willmar Schwabe GmbH & Co. KG, manufacturer of EPs 7630, who also provided the original patient data for the meta-analyses. Main characteristics of the trials are shown in Table 1.

**Table 1:** Main characteristics of eligible trials

Indication	Study, reference	Treatment duration, daily dose	Maximum daily paracetamol dose allowed^%^	Primary efficacy outcome measure ^+^	Number of patients^#^	
					EPs 7630	Placebo
Acute tonsillopharyngitis	A Timen et al., 2015 [60]	6 days: Day 1–2: 20 drops hourly while awake, Day 3–6: 3 × 20 drops	Up to 3 × 500 mg on days 0 to day 4	Response: TSS (7 symptoms) ≤ 4 points at day 4	40	38
	B Berezhnoi et al., 2016 [55]	6 days: 3 × 20 drops		TSS (5 symptoms): difference baseline – day 4	60	64
	C Bereznoy et al., 2003 [56]	6 days: 3 × 20 drops		TSS (5 symptoms): difference baseline – day 4	73	70
Acute bronchitis	D Kamin et al., 2010a [58]	7 days: Age ≤ 6: 3 × 10 drops Age 7–10: 3 × 20 drops	Age ≤ 6: 3 × 250 mg Age 7–10: 3 × 500 mg	BSS: difference baseline – day 7	25^§^	24^§^
	E Kamin et al., 2010b [59]	7 days: 3 × 20 mg (tablets)	3 × 500 mg	BSS: difference baseline – day 7	32^§&^	31^§^
	F Kamin et al., 2012 [57]	7 days: Age ≤ 6: 3 × 10 drops, Age 7–10: 3 × 20 drops	Age ≤ 6: 3 × 250 mg Age 7–10: 3 × 500 mg	BSS: difference baseline – day 7	35^§^	31^§^

^%^ Only allowed on days with fever ≥38.5 °C

^+^ *TSS* Tonsillopharyngitis Symptom Score (total score), *BSS* Bronchitis Symptom Score (total score)

^#^ Primary efficacy analysis data set (full analysis set)

^§^ Only children 6–10 years of age

^&^ Only children randomised to 3 × 20 mg/day

Three studies (A, B and C) [55, 56, 60] investigated the efficacy and safety of EPs 7630 in ATP according to similar protocols. They included girls and boys between 6 and 10 years of age who presented with signs and symptoms of ATP persisting for ≤48 h. A negative rapid antigen-detection test for β-haemolytic streptococci was required in order to exclude children with GAS. Moreover, a minimum total score of 6 points on a 7-item Tonsillopharyngitis Symptom Scale (TSS; symptoms assessed: dysphagia, sore throat, salivation, redness, coating left, coating right, fever; total score range 0–21 points) in trial A, or of 8 points (5-item TSS; symptoms assessed: dysphagia, sore throat, salivation, redness, fever; total score range 0–15 points) in trials B and C applied to assure sufficient symptom severity. The scheduled treatment period of all trials in ATP was 6 days.

In AB, 3 trials (D, E, and F) performed according to protocols with similar target populations, study procedures, schedules, and assessments were identified that investigated male and female children and adolescents up to the age of 18 [57–59], The subset of children between 6 and 10 years of age was included into our meta-analysis. In all 3 studies, eligible participants had to present with signs and symptoms of AB persisting for ≤48 h prior to inclusion. For inclusion, the protocols also required a total score ≥ 5 points on the 5-item Bronchitis Severity Scale (BSS; symptoms assessed: cough, sputum production, rales at auscultation, chest pain during coughing, dyspnea; total score range: 0–20 points) [70]. Like in all trials in ATP, the participants of studies D and F received EPs 7630 as solution. Trial E was a dose finding study whose participants were randomised to receive tablets of 3 × 10, 3 × 20, or 3 × 30 mg/day EPs 7630 or placebo. For the present meta-analysis, only children who received the marketed dose of 3 × 20 mg/day or placebo were considered. All trials in AB had a treatment duration of 7 days.

The total number of children analysed was 265 for EPs 7630 (ATP: 173; AB: 92) and 258 (ATP: 172; AB: 86) for placebo.

The protocols of all eligible studies allowed paracetamol suppositories and tablets as supporting medication in case of fever ≥38.5 °C. In the trials in ATP, paracetamol was allowed on days 0 through 4 after the start of randomised treatment, up to a maximum daily dose of 3 × 500 mg. The protocols of studies D and F permitted up to 3 × 250 mg suppositories per day for children ≤6 years of age. For older children, 3 × 500 mg tablets were permitted in study D. As concerns study F, 3 × 500 mg suppositories were allowed for children between 7 and 12 years and 3 × 500 mg tablets for older children. In study E, 3 × 500 mg tablets per day were allowed for children of any age. All administered doses had to be documented. Any other concomitant medication with potential impact on study outcomes and/or on the course of ATP or AB (e. g., antibiotic treatment) was forbidden.

Table 2 shows the basic demographic data of the study participants. Patient age was comparable for EPs 7630 and placebo, and also for ATP and AB. Coincidentally, more children randomised to EPs 7630 were female (as compared to male), whereas more children who received placebo were male, for both ATP and AB.

**Table 2:** Demographic data

Indication	Study, reference	Treatment	Age (years) mean ± SD, range	Sex	
				Female	Male
Acute tonsillopharyngitis	A Timen et al., 2015 [60]	EPs 7630 (*n* = 40)	7.4 ± 1.2, 6–9	26 (65.0%)	14 (35.0%)
		Placebo (*n* = 38)	7.7 ± 1.2, 6–9	18 (47.4%)	20 (52.6%)
	B Berezhnoi et al., 2016 [55]	EPs 7630 (*n* = 60)	7.6 ± 1.1, 6–9	29 (48.3%)	31 (51.7%)
		Placebo (*n* = 64)	7.4 ± 1.2, 6–9	28 (43.8%)	36 (56.3%)
	C Bereznoy et al., 2003 [56]	EPs 7630 (*n* = 73)	7.6 ± 1.3, 6–9	40 (54.8%)	33 (45.2%)
		Placebo (*n* = 70)	7.5 ± 1.1, 6–9	30 (42.9%)	40 (57.1%)
Acute bronchitis	D^§^ Kamin et al., 2010a [58]	EPs 7630 (*n* = 25)	7.8 ± 1.3, 6–10	14 (56.0%)	11 (44.0%)
		Placebo (*n* = 24)	7.9 ± 1.7, 6–10	11 (45.8%)	13 (54.2%)
	E^§^ Kamin et al., 2010b [59]	EPs 7630 (*n* = 32)	8.2 ± 1.3, 6–10	16 (50.0%)	16 (50.0%)
		Placebo (*n* = 31)	7.9 ± 1.5, 6–10	15 (48.4%)	16 (51.6%)
	F^§^ Kamin et al., 2012 [57]	EPs 7630^&^ (*n* = 35)	7.7 ± 1.3, 6–10	21 (60.0%)	14 (40.0%)
		Placebo (n = 31)	7.4 ± 1.3, 6–10	16 (51.6%)	15 (48.4%)
Pooled data (acute tonsillopharyngitis + acute bronchitis)		EPs 7630 (*n* = 265)	7.7 ± 1.2, 6–10	146 (55.1%)	119 (44.9%)
		Placebo (*n* = 258)	7.6 ± 1.3, 6–10	118 (45.7%)	140 (54.3%)

^§^ Only children 6–10 years of age

^&^ Only children randomised to 3 × 20 mg/day

The trials in ATP were performed according to an adaptive, group-sequential design whereas the studies in AB used a design with one adaptive interim analysis. Measures implemented to control bias on the individual study level included the recruitment of consecutive eligible children whose legal representatives provided informed consent, blinding of the investigational treatments (including blinded outcome assessments) as well as randomised assignment to EPs 7630 and placebo. We contacted the manufacturer of EPs 7630 to assure that all randomised, placebo-controlled studies investigating the administration of the product to children with ATP or AB had been identified. Publication bias was ruled out by including into our analyses all studies performed in the indications and population of interest. Since all studies reported data for the pre-defined outcome measures of interest, our analyses were not at risk of within-study selective reporting.

### Main results of eligible trials

Studies A – C investigated the efficacy of EPs 7630 in the symptomatic treatment of ATP. Main efficacy assessments were based on TSS total score change. In study A, the primary outcome measure was a responder rate where response was defined as a TSS total score ≤ 4 points at day 4 of randomised treatment. 90% of the 40 children in the EPs 7630 group responded, compared to 44.7% of the 38 children who received placebo (*p* < 0.001; Fisher’s exact test). The primary outcome measure for treatment efficacy in studies B and C was the change of the TSS total score between baseline and treatment day 4. In study B, TSS total score decreases of 6.7 ± 2.7 (mean ± SD) and of 3.3 ± 4.2 points were observed for EPs 7630 and placebo, respectively (95% CI for mean value difference: [2.0; 5.2 points]). In study C, children treated with EPs 7630 showed a TSS total score decrease by 7.1 ± 2.1 points, compared to 2.5 ± 3.6 points in the placebo group (95% CI for mean value difference: [3.3; 5.7 points]).

The efficacy of EPs 7630 in the symptomatic treatment of AB was investigated in studies D – F. The pre-defined primary efficacy outcome measure in all trials was the BSS total score change between baseline and day 7. The BSS total score was reduced by 3.4 ± 1.8 points for EPs 7630 and by 1.2 ± 1.8 points for placebo in trial D (*p* < 0.001; treatment main effect from an analysis of covariance model that also included a main effect for centre and the BSS baseline value as a covariate; applies to studies D through F), by 4.4 ± 2.4 points for EPs 7630 3 × 20 mg/day and by 3.3 ± 2.6 points for placebo in trial E (p < 0.001), and by 4.4 ± 1.6 points for EPs 7630 and by 2.9 ± 1.4 points for placebo in trial F (p < 0.001). For AB, these results apply to the primary analyses according to the study protocols and include patients in the entire age range eligible for trial participation, i. e., 0–18 years for studies D and F, and 6–18 years for study E. Separate analyses for the age range of 6–10 years considered in our meta-analysis were not performed.

### Paracetamol consumption

**Fig. 2:**
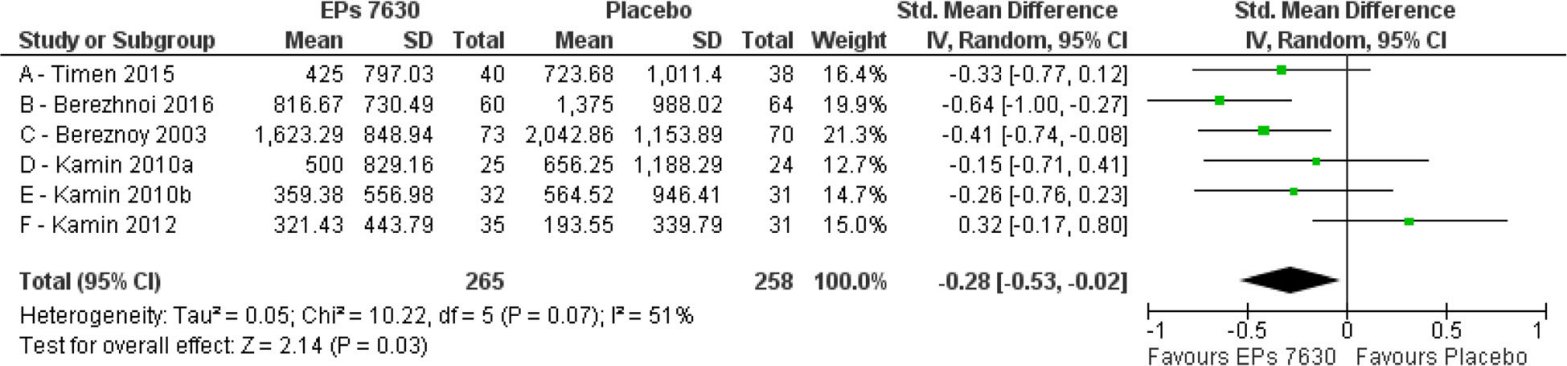
Average cumulative paracetamol use (mg) – standardised effect size (Hedges’ g)

Figure 2 shows the main results of the meta-analysis of cumulative paracetamol use during study participation. The average cumulative paracetamol doses administered in both treatment groups in AB were generally somewhat lower than in ATP.

Out of the 6 studies included into the analysis, 5 showed a lower average cumulative paracetamol dose in the EPs 7630 group and 1 showed a lower dose in the placebo group. On the study level, treatment group differences ranged between a 558 mg decrease and a 128 mg increase in average paracetamol dose in the EPs 7630 group as compared to placebo, with a weighed mean difference of 244 mg across all studies favouring EPs 7630.

The meta-analysis shows that the average cumulative paracetamol dose was significantly lower in the EPs 7630 group as compared to placebo (Hedges’ g: -0.28; 95% CI: -0.53; − 0.02; *p* = 0.03). Significant differences favouring EPs 7630 were also observed in 2 out of the 6 studies, B and C (*p* < 0.05). The diagnostic tests performed in the context of the meta-analysis indicate moderate heterogeneity of the treatment effects observed in the analysed trials (I^2^ = 51%; χ^2^-test: *p* = 0.07) [71], which was mainly attributable to the discrepancy between the results of study F and those of all other trials.

### Inability to attend school

**Fig. 3:**
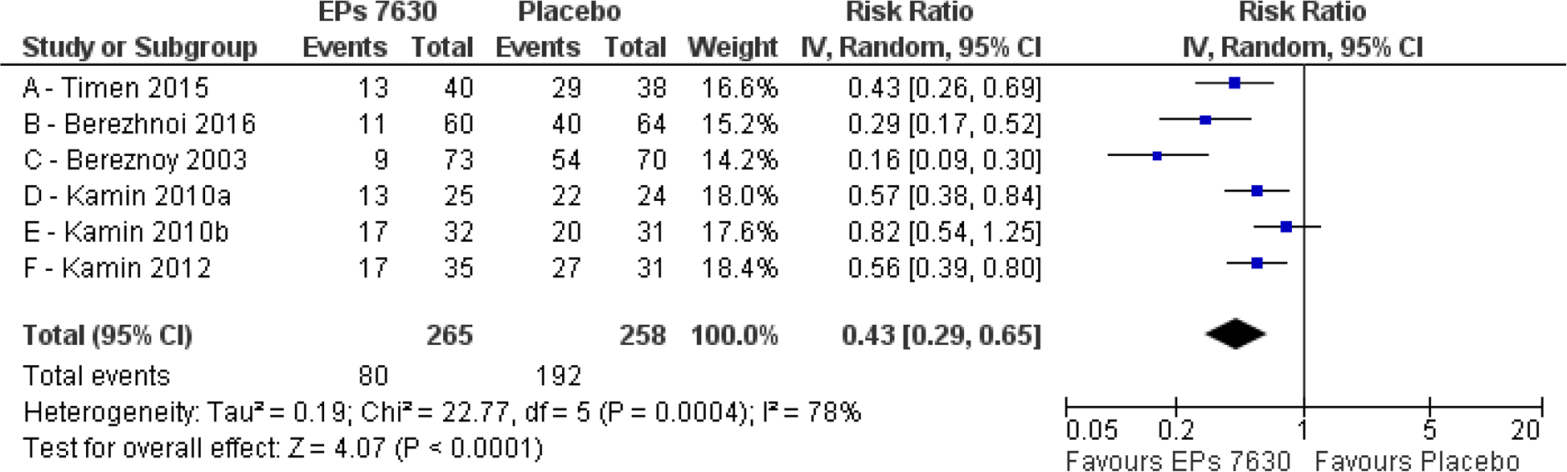
Number of children unable to go to school at day 6 (acute tonsillopharyngitis) or day 7 (acute bronchitis) after start of treatment

The number of children who were unable to attend school at treatment end (identified as ‘events’) was significantly higher in the placebo group as compared to EPs 7630 in 5 of the 6 studies included (Fig. 3). In the pooled data set, the observed event rates were 30.2% (80/265 children) and 74.4% (192/258 children) for EPs 7630 and placebo, respectively, corresponding to a meta-analysis risk ratio of 0.43 (95% CI: 0.29; 0.65; *p* < 0.001) favouring EPs 7630.

In this meta-analysis, an I^2^ value of 78% (χ^2^-test: *p* < 0.001) indicates substantial heterogeneity between the trial results [71]. Heterogeneity was, however, caused by the magnitude, not by the direction of the treatment effects, since advantages for EPs 7630 over placebo were observed in each of the included trials, and 5 out of the 6 studies showed a significant effect even when considered individually.

## Discussion

Acute RTIs, such as AB or ATP, are usually not associated with febrile temperatures in a range that requires antipyretic treatment [2, 8, 72]. On the contrary, medically unnecessary antipyresis may interfere with the organism’s adaptive response to viral infection, may thus even prolong illness (although it does not prolong the persistence of fever) and should therefore be avoided [2, 4, 9, 73]. Consequently, current guidelines recommend against using antipyretic agents with the sole aim of reducing body temperature in children with fever [74].

Although paracetamol, the most widely used antipyretic medication in children, has long been recognized as safe and efficacious when used in the recommended dose range [46–48], it has nevertheless also been associated with adverse effects, some of which may be potentially serious although rare. Probably the most serious complication in the paediatric setting is hepatotoxicity caused by accidental overdose [48, 75, 76]. Medication errors involving paracetamol are not surprising, since the drug is so widely used in children and is available in so many different formulations. Parents may sometimes not even be aware that they are administering paracetamol [49, 77]. Moreover, there is convincing evidence that paracetamol use by infants, or even by expecting women, may increase the risk of asthma, rhinoconjunctivitis, and eczema in children [50–52, 78–80], although a recent meta-analysis shows that childhood asthma may also be related to respiratory infections during infancy rather than to paracetamol administered to treat them [81].

Antipyretic drugs such as paracetamol are generally found to improve the comfort of the ill child, and thus they also reduce the strain on parents and health care professionals [13, 14, 82]. This may be the reason why such drugs are so widely used, even though antipyresis has been shown to be unhelpful in trivial viral infections [2, 4, 9]. Indeed, improving the over-all well-being of the ill child is a primary objective of supportive therapy [2]. Since commonly used antipyretic drugs, including paracetamol, exert their beneficial influence in trivial infections mainly through their analgesic rather than through their antipyretic effect [2, 4], it may be preferable to revert to treatments that provide symptom control, improve the child’s over-all comfort, and accelerate recovery without causing unneeded antipyresis.

The analyses of the symptom scales pre-defined as primary outcome measures of the trials included into this meta-analysis confirm that EPs 7630 is efficacious in reducing the symptom burden and in accelerating recovery in children suffering from AB or ATP [55–60]. This meta-analysis adds to the existing evidence by demonstrating that EPs 7630 also reduces the use of paracetamol and enables ATP- and AB-affected children to return to school earlier, thus effectively shortening the course of the disease.

The protocols of the studies included into our meta-analysis allowed paracetamol co-medication only in case of fever ≥38.5 °C. Although the studies did not explicitly assess whether the drug was administered for analgesia or for antipyresis, the permission to use paracetamol was associated with the child’s temperature level, and it may thus reasonably be assume that the decision of the parents to administer the drug was primarily motivated by the reduction of fever. Since EPs 7630 has no known direct antipyretic effect, the fact that the cumulative paracetamol dose in the EPs 7630 group was systematically lower than in the placebo group may indicate that the children treated with the herbal product were less febrile, not as a result of antipyretic treatment, but because of a more favourable course of their infection. This is also consistent with the observation that children exposed to EPs 7630 recovered from RTI associated symptoms more rapidly.

Between-study heterogeneity observed in our meta-analyses was mainly attributable to differences in the magnitude of the effect favouring EPs 7630 rather than in the direction of the effect. For paracetamol consumption, 5 out of 6 trials in the meta-analysis showed lower antipyretics use in the EPs 7630 group as compared to placebo, with descriptively significant advantages for EPs 7630 in 2 of these trials. For inability to attend school, all included studies favoured EPs 7630, and 5 out of 6 showed descriptively significant advantages for the herbal product. The meta-analyses also indicate, however, that the effect sizes of EPs 7630 over placebo were somewhat more pronounced in the studies performed in ATP than in those in AB, which also contributed to the observed heterogeneity.

A methodological weakness of the study is that the observed use of paracetamol could not be intraindividually correlated to actual body temperature since temperature measures were not reported systematically. Instead, we had to rely on the instruction given to the parents, to administer paracetamol only in case of a body temperature of 38.5 °C or higher. Another weakness lies in the comparatively small number of subjects available for meta-analysis, notably in AB, where only the subsets of children between 6 and 10 years of age were eligible for analysis. It is noteworthy, however, that the sample size of each study was planned according to statistical considerations, and that it proved to be sufficient for demonstrating superiority of EPs 7630 over placebo for the pre-defined primary outcome measure. Moreover, particularly in a vulnerable patient population like children, it can hardly be justified from an ethical perspective to include more subjects into a placebo-controlled trial than the minimum number required for providing acceptable statistical power for achieving the primary study objective.

## Conclusions

In conclusion, randomised, controlled trials confirm that EPs 7630 reduces the severity and duration of disease associated symptoms in children with non-GAS ATP and AB. Our results support and add to these findings by showing that children treated with EPs 7630 required less paracetamol co-medication and were able to return to school earlier. We consider both observations to be patient-relevant benefits since EPs 7630 ameliorated the unpleasant effects of the febrile response, achieved symptomatic improvement and accelerated the restauration of normal functioning without potentially counterproductive antipyresis, also reducing patient risk by avoiding the potentially harmful side effects of paracetamol.

## Abbreviations

AB: Acute bronchitis
ATP: Acute tonsillopharyngitis
BSS: Bronchitis Severity Scale
CI: Confidence interval
FDA: Food and Drug Administration
GAS: Group A streptococcus
OTC: Over-the-counter
RTI: Respiratory tract infection
TSS: Tonsillopharyngitis Symptom Scale

## Footnotes

1 EPs® 7630 is the active ingredient of the product Umckaloabo® (ISO Arzneimittel, Ettlingen, Germany).
